# Sub-patterns of food consumption and hyperglycemia in Mexican young people: a study by factor analysis

**DOI:** 10.3402/fnr.v60.30185

**Published:** 2016-02-26

**Authors:** Juan Antonio Córdova Hernández, David del Valle Laveaga, Juan Manuel Muñoz Cano

**Affiliations:** Health Sciences Academic Division, University Juarez of Tabasco, Tabasco, Mexico

**Keywords:** dietary patterns, eating habits, modern foods, prediabetes, diabetes

## Abstract

**Background:**

The student population that is admitted to the University Juarez of Tabasco has poor healthy eating habits. Fasting glucose ≥5.6 mmol/L was found in 10% of the students.

**Objective:**

We wanted to identify the sub-pattern of their eating habits that could explain the hyperglycemia.

**Design:**

A questionnaire on the feeding habits was applied to 3,559 first-year students, who were subjected to a blood analysis to determine biochemical markers in 2011. Based on the obtained questionnaire data, the factorial analysis was used for the statistical analysis. The Kaiser–Meyer–Olkin measure for sampling adequacy was used for validation. To determine eating habits, Varimax normalization with Kaiser was used.

**Results:**

The number of students with euglycemia was 3,138, including 366 with values for prediabetes, and 55 with values for diabetes. After normalization using Varimax rotation with Kaiser, component 1 of participants with euglycemia included eight foods. The number of foods in component 1 of those participants with prediabetes was seven, and it diminished to four in those with fasting glucose >7 mmol/L.

**Conclusions:**

It was found that glucose levels increase in direct relation to the diminution in the number of selected foods.

The increase in type 2 diabetes mellitus (DM2) is preceded by the increase in average blood glucose values in the world (1). This explains why DM2 is detected in younger people more than in the past, and not only as a result of modifications in the diagnostic criteria like prediabetes definition, that is the present concept for fasting glucose levels of ≥5.5 mmol/L. Although timely screening is recommended starting at the age of 45 years (2), the DM2 cases, which were once infrequent during childhood and adolescence, have become more common, even though the diagnostic criteria for this age group are still being discussed (3).

Even though DM2 is considered a group of diseases resulting from environmental and genetic factors and used to be associated with obesity, it is found more frequently in people that have altered their eating habits (4–6). The change in eating habits from those based on traditional ethnic foods toward modern industrialized foods has been correlated with a greater probability of developing DM2 (7), as well as with other non-transmissible diseases such as hypertension, atherosclerosis, vascular disease, chronic renal disease, non-alcoholic non-autoimmune liver cirrhosis, and several types of cancer (8, 9).

The University Juarez of Tabasco (UJAT), among its enrollment requirements, requests that a complete medical history, complete laboratory blood tests, including glucose, triglycerides, and cholesterol determination, be performed routinely. In this way, in 2011, it was found that 10% of the enrolled population had fasting glucose levels of 5.6–6.9 mmol/L, and these values were ≥7 mmol/L in 1%. It was also found that these anomalies in biomarkers did not correlate with either the body mass index (BMI) or other anthropometric indices (10).

To assess eating habits and relate them to the encountered biochemical anomalies, as well as to elaborate an educational strategy with UJAT students, in 2011, we applied a questionnaire on their eating habits to 3,559 first-year students. The classification of the eating habits according to the healthy nutrition index revealed that only 0.1% of the students qualified for the optimal level, ‘healthy eating’, whereas 19.7% had habits considered as ‘need of changes’ and 80.2% as ‘poorly healthy’ (11).

In that search, it was found that fasting glucose ≥5.6 mmol/L in 10% of students with pattern of food consumption qualified ‘need of changes’ as in ‘poorly healthy’, so it was deemed necessary to perform a more in-depth study. We chose for statistical analyses the principal components methodology (12), through a factorial analysis of the results of eating habits reported for the month before the questionnaire was applied. The aim was to identify a sub-pattern of food consumption that could explain why there was a group of freshman students with glucose values above the cutoff limit for prediabetes independently from their BMI and their healthy nutrition index group. Based on the aforementioned, the objective of this study was to identify a sub-pattern related to a greater possibility of presenting fasting glucose levels considered for adults as prediabetes (5.6 to 6.9 mmol/L) or diabetes (≥7 mmol/L).

## Methods

### Type of study

**Fig. 1:**
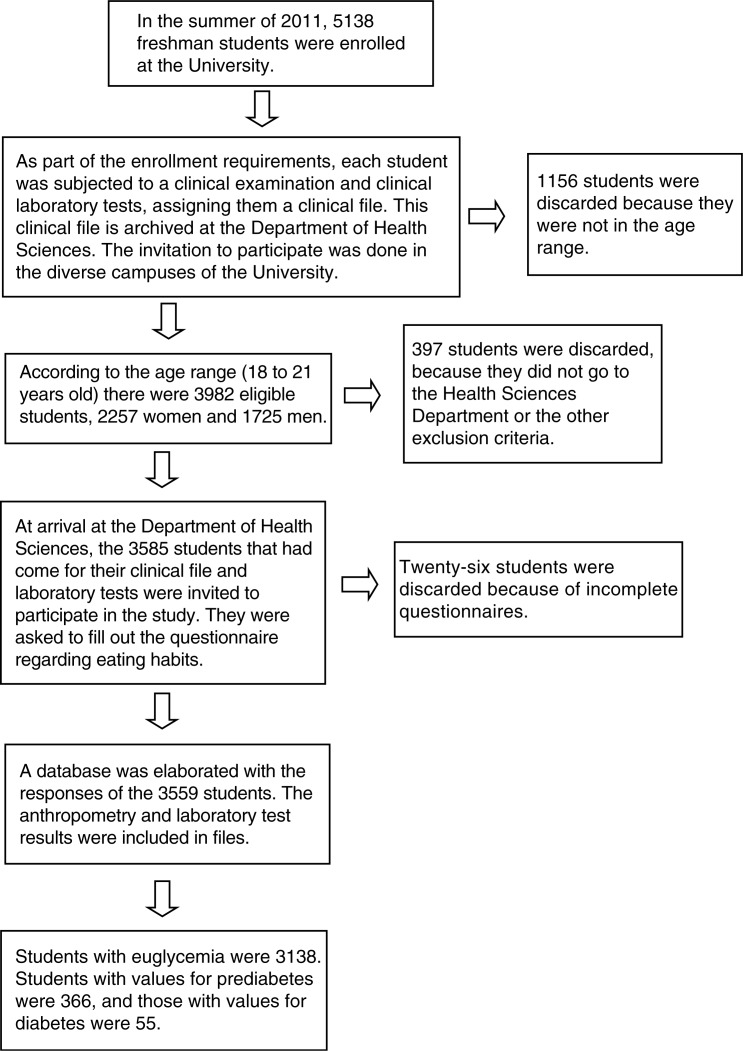
Selection of the participants.

To analyze the characteristics of the dietary patterns of first-year students at the UJAT, we performed an observational, analytical, cross-sectional cohort study. As part of the enrollment process, students are subjected to blood sampling to evaluate biochemical markers and for the elaboration of a clinical file; this is made at the clinical analyses laboratory of the University and at the Clinical Center of the Health Sciences division. Those that were subjected to this health process from September to November 2011, 5,138 freshman students, were invited to participate in the present study. Exclusion criteria included those students being treated for diabetes mellitus, or taking lipid-lowering drugs, pregnant, out of the 18- to 21-year-old range, or refused to participate. A total of 3,559 students participated, 2,040 women and 1,519 men. We chose the age range from 18 to 21 years with mean 18.65±0.9 years for the sake of higher homogeneity, although the UJAT enrolls students of other ages (Fig. 1).

### Dietary patterns

Dietary pattern was defined as the preference of people for determined products. Dietary patterns were identified by applying the CDC-FFQ questionnaire (13) in its Spanish version and adapted for its use in Mexico, including fresh produce and food of the region (11, 14). Responses regarding the month previous to the application of the questionnaire were the ones considered for the purposes of the present study. To identify the pattern of eating habits, we used the classifications of traditional, traditional industrialized, and modern industrialized (15). Traditional foods are those consumed ‘sometimes raw, without any processing aside from its collection, hygiene considerations, and selection’. Traditional industrialized foods, like corn tortillas, were ‘part of the diet of Mexicans before the XX century’, whose supply has changed from artisanal to industrial and large-scale production. The modern industrialized foods, like margarine and dressings, ‘can be found in a single food or as indivisible mixture’ (16). For the analysis of principal components, 27 categories were constructed (Table 1).

**Table 1:** Category of food groups

Categories	Groups of food
Traditional	Red meats, tubercles, poultry, vegetables, fish, cereals, leguminose, eaten fruits, hot food, dried fruits, eggs
Traditional industrial	White bread and tortillas, coffee and infusions, fried food
Modern industrial	Cold cuts, sugar added, dairy, pastry, dressings, fatty food, pastas, breakfast cereals, juices, alcoholic beverages, high-protein beverages, sodas

### Anthropometry

Those that agreed to participate in the study were asked to allow the procurement of weight and height. Seven students (six majoring in nutrition and one in nursing) collaborated in procuring the anthropometric data; these students were previously trained to standardize the procurement of data for these procedures. To determine the BMI, a 200-kg capacity clinical balance with a stadiometer was used (Básculas Nuevo León^®^, Mexico). The balance was calibrated daily during the whole time during which students were received.

The BMI was calculated according to the mathematical expression mass/height^2^=kg/m^2^ and interpreted according to World Health Organization (WHO) guidelines. For women taller than 1.5 m and men taller than 1.6 m: low weight ≤18.49, eutrophic 18.5–24.99, overweight 25–29.99, and obesity ≥30. For women below 1.49 m and men below 1.59 m: low weight ≤18.49, eutrophic 18.5–22.99, overweight 23–24.99, and obesity ≥25.9.

### Biochemical parameters

The personnel of the clinical analyses laboratory of the UJAT obtained 12-h fasting blood samples from the participants. Sterile equipment was used, and blood was collected in Vacutainer^®^ Serum tubes (Becton Dickinson, Franklin Lakes, NJ, USA). Once in the laboratory, the serum was obtained by centrifuging at 3,500 *g* during 3 min in less than 30 min after blood sampling to avoid glycolysis. In that blood fraction, glucose (GA), total cholesterol (TC), low-density lipoproteins (LDL), high-density lipoproteins (HDL), and triglycerides (TG) were measured. All these parameters were determined by means of dry analytical methodology using automated VITROS^®^ 250 equipment (Ortho-Clinical Diagnostics Johnson & Johnson, Rochester, NY, USA).

Glucose was assessed according to the American Diabetes Association (ADA) criteria. Euglycemia<5.5, fasting-altered glucose (prediabetes) from 5.6 to 6.9 mmol/L, and diabetes ≥7 mmol/L. Clinical criteria for fasting glucose were euglycemia when glucose ≤5.5 mmol/L, prediabetes if glucose was >5.5 but <6.9 mmol/L, and diabetes if glucose was ≥7 mmol/L.

To assess serum lipids, criteria of the National Cholesterol Program Adult Treatment Panel III, 2005, were used. Triglycerides (TG) were considered desirable at ≤1.68 mmol/L, borderline from 1.69 to 2.25 mmol/L, and high ≥2.26 mmol/L. TC values were considered desirable at ≤4.39 mmol/L, borderline from 4.4 to 5.16, and high ≥5.17. HDL cholesterol was considered optimal if ≥1.55 mmol/L, risky if ≤1.03 in men and 0.9 in women. LDL cholesterol was calculated using the Friedewald formula: LDL (mmol/L)=total cholesterol – (TG/5) – HDL, where optimal is ≤2.83 mmol/L, borderline from 2.84 to 3.35 mmol/L, high ≥3.36 mmol/L.

### Statistical analysis

Version 21.0 of the IBM SPSS (Chicago, IL, USA) was used for data processing. Statistical tests of central tendency were made for biochemical marker's data processing. A diagram of boxes and whiskers was made to learn the position and variability of the distribution of frequencies of glycemia, the dependent variable. A chi-square test was used to assess differences among glucose values and their association with gender and body mass volume. To determine the predictive power of each anthropometric index, we performed studies on the significantly different probability (SDP) and Z score with the Minitab^®^ software (Minitab Inc., USA), which was considered significant at ≥1.96.

To determine dietary patterns, the independent variable, the principal components methodology was used. It was found that the first component explained 30.38% of the variance, and six factors were analyzed that corresponded to 56.25% of the accumulated variance (cutoff eigenvalue ≥1). Before determining the scores and factorial loads, the Kaiser–Meyer–Olkin (KMO) sampling adequacy test was performed, considering significant value to be KMO >0.9 with *p*<0.01. To explain better the results, values ≤0.59 were eliminated. Once the principal components were obtained and labeled, the rotation method of the Varimax normalization with Keiser was used to diminish the load of factors and to find a new group of variables that would explain better the model.

### Ethical considerations

The study complied with the Helsinki Declaration of the World Medical Association and with the legislation established by the General Law for Research in Health of Mexico. The project was registered at the Research Division of UJAT with code TAB-2010-C19-144012. Although the research is considered ‘without risk’, each participant signed an informed consent. Each participant was briefed on the objectives of the research and was given the possibility of not responding to the questionnaire on eating habits.

## Results

**Fig. 2:**
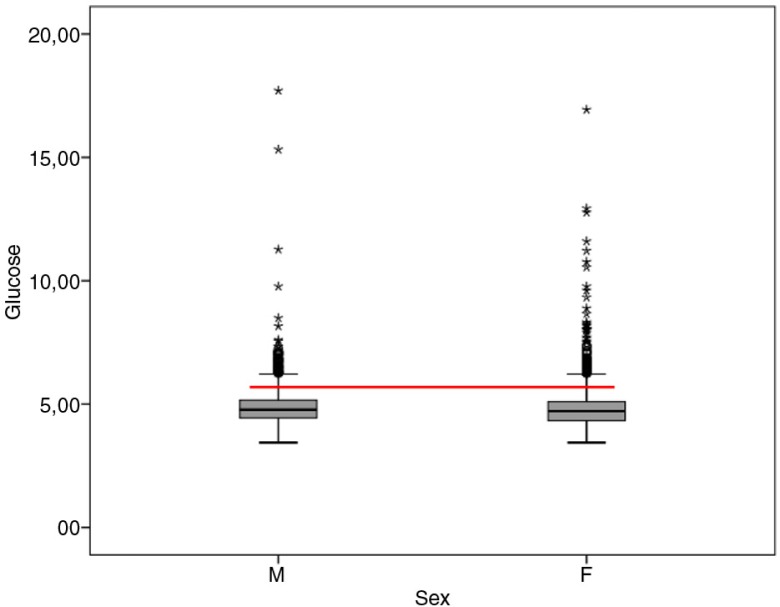
Levels of fasting glucose. Glucose in mmol/L. The horizontal line separates the students with euglycemia ≤5.5 mmol/L (under) from those with hyperglycemia values ≥5.6 mmol/L (up). Chi-square test results for gender and fasting glucose had a value of 1.783 with *p*=0.410, which shows that there was no association between gender and glucose.

More women (6.3%) with low weight than men (3.2%), as well as more women with eutrophic weight (50.9%) than men (31.1%), were found: there were more overweight and obese men (44.3 and 21.3%) than women (27.2 and 15.1%). Assessment of biochemical markers revealed that glucose values were similar for women and men (women mean 4.8 mmol/L±0.85, 95% CI 4.79–4.86; men mean 4.87 mmol/L±0.8, 95% CI 4.83–4.91) (Fig. 2). We measured the mean and median fasting glucose levels for each gender. For women, the mean value was 87 and the median was 85 with an asymmetry value of 3.9. For men, the mean was 87.84, and the median was 86 with an asymmetry value of 5.14. Although values are not identical, means and medians are very close, and thus, distribution of data is symmetrical.

**Fig. 3:**
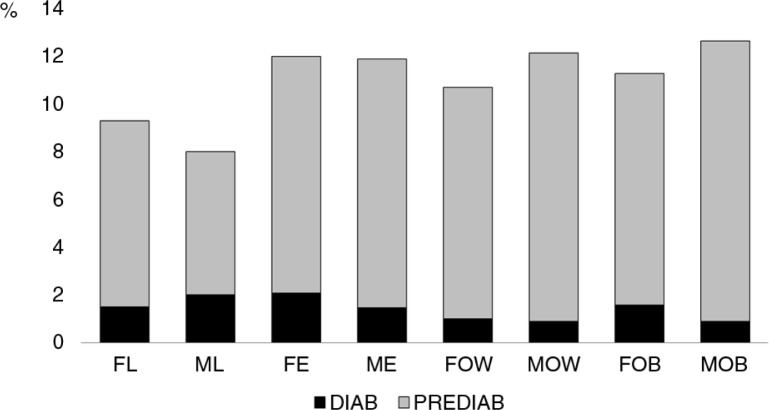
Fasting glucose levels and body mass in%. FL, females in low weight; ML, males in low weight; FE, females in eutrophic weight; ME, men in eutrophic weight; FOW, females in overweight; MOW, males in overweight; FOB, females in obesity; MOB, males in obesity. Gray, 5.6–6.9 mmol/L prediabetes (PREDIAB). Black, ≥7 mmol/L diabetes (DIAB).

No correlation existed between BMI and fasting glucose values, as there were similar percentages of students with prediabetes and diabetes for each category of body mass, in average 10.3 and 1.5%, respectively, for the studied population. When analyzing fasting glucose values, similar percentages of prediabetes and diabetes were found for men and women, independently from the BMI. SDP of 26.62 was found in women and of 21.28 in men. A Z-score of 0.33 and 0.26, respectively, was attained when comparing low-weight to obese individuals. The results of the statistical test provide support to the notion that both categories are similar for men and women (Fig. 3).

Triglycerides determination was mean 1.39 mmol/L±0.738 in men and mean 1.23 mmol/L±0.567 in women. LDL was mean 1.19 mmol/L±0.99 in men and mean 1.27 mmol/L±0.98 in women. No significant differences existed between genders. For the 3,556 participants, a correlation was found between fasting glucose levels and those of triglycerides (*r*=0.15 and *p*=0.0001); glucose and TC (*r*=0.46 and *p*=0.0001); glucose and LDL (*r*=0.48 and *p*=0.0001).

We performed Cronbach's alpha test for internal consistency of the 198 elements of the questionnaire; the value was 0.93. To determine the dietary patterns, a factor analysis test was performed. There was enough correlation among groups of foods to make a principal components analysis with a KMO measure of sampling adequacy of 0.92 and *p*=0.0001. Since no significant differences were found in the initial analysis of the results from women with respect to the analysis of dietary patterns of men, the tests were performed only with the general database.

The first component was labeled as modern industrialized. Although the first group of foods corresponded to red meats, with a 0.76 value, the next ones were of modern industrialized: cold cuts, milk products, industrialized pastry, dressings and pickles, industrialized bread, pastas; foods with high fat and sugar content; tubercles, mainly potatoes; and poultry, with the least component value, 0.62. The second component in general included alcoholic beverages, protein-based liquid foods, and sodas of all types.

When applying the rotation test of the Varimax normalization with Kaiser, the group of cold cuts increased its value to 0.8, followed by red meats (0.75), fried food (0.75), pastas (0.67), fatty food (0.62), and sugar-added food (0.61). This process left out poultry, breakfast cereals, tubercles, with values between 0.56 and 0.49. This confirmed that the dietary pattern was mainly modern industrialized.

### The sub-pattern of food consumption

As there was interest in finding a sub-pattern of food consumption that would allow explaining the high frequency of hyperglycemia found in this age group, the participants were separated according to three clinical categories. The group with euglycemia, 3,138 (88.2%) students, presented a Varimax with Keiser normalization measure of 0.92 and *p*=0.0001. Applying the tests to the students with glucose 5.6 to 6.9 mmol/L, 366 participants (10.3% of the studied population), the Varimax with Keiser normalization measure was of 0.911 and *p*=0.0001. In those with glucose >7 mmol/L, comprised 55 students (1.5% of the population), the Varimax with Kaiser normalization value diminished to 0.698, but retained significance at *p*=0.0001.

According to the first component (Table 3), the dietary pattern of the participants with euglycemia is similar to that of the general population as they comprise most of the students. After Varimax normalization with Kaiser, it was found that component 1 of the participants with euglycemia included 8 foods. The number of foods in component 1 in those participants with prediabetes was 7, and it decreased to 4 in those with fasting glucose >7 mmol/L. In the first component of those included in the prediabetes category, labeled also as ‘modern industrialized’, there was only one traditional, red meats, and one traditional industrialized, white bread. For those in the diabetes category, a higher value was found for cold cuts, 0.83, and was accompanied only by fatty food (0.75), fried food (0.67), and white bread (0.62), indicating an increase in food with larger amounts of calories.

The second component for those in the euglycemia group was labeled ‘liquid foods and beverages’ and was different for the prediabetes group (traditional food) and those of the diabetes group. The second component included fruits and therefore could be considered healthier; however, by including juices and dairy products, the supply of simple sugars and cholesterol increased. For the third component, there were no results for the group in euglycemia. In the prediabetes group, juices were found in the first place followed by legumes in the second place. Although this second food is traditional, juices constitute a new dietary pattern, but are not recommended due to their high sugar content. This made it difficult to label this component. The third component of the diabetes group was labeled sugar and alcohol. The foods included in this component have a high content of added sugar.

## Discussion

As has been demonstrated in other populations, it is not necessary to develop a large body mass to reach fasting hyperglycemia (17). The high proportion, almost half of the participants, that presented increases in TC and LDL, as well as a diminution in HDL (Table 2), explains the possible correlation between hyperglycemia and hyperlipidemia. Although some of the high values could be transient, once analyzed in the context of the large proportion of overweight–obesity and the correlation between biomarkers and anomalies, it must be considered that this group of young adults, in their late teens, is in average at risk levels, which will increase with age and lead to a greater load of non-transmissible diseases.

**Table 2:** Levels of biochemical markers

	Clinical levels *n* (%)		
	
Biomarker	Desirable	Borderline	Risk
Glucose	3,138 (88.2)	366 (10.3)	55 (1.5)
Triglycerides	2,876 (80.8)	440 (12.4)	243 (6.8)
Total cholesterol	1,931 (54.3)	1,104 (31)	524 (14.7)
LDL cholesterol	1,685 (47.3)	670 (18.8)	1,204 (33.8)
HDL cholesterol^a^	695 (19.5)	2,094 (58.8)	770 (21.6)

For glucose: borderline=prediabetes; risk=diabetes.

a For HDL cholesterol, risk means values below the cutoff limit.

### The sub-pattern of food consumption

Similar to other studies on adolescents and young Latin American university students (18, 19), the selection of foods was limited and directed toward those classified as modern industrialized. The predominant eating pattern was of high amounts of processed meats, industrialized pastry, fried foods, refined flour and sugar-containing bread; ingestion of vegetables, fruits, and fish was small and that of legumes, particularly black beans, was moderate. Although no physical activity habits were determined as done in other classifications, this pattern of eating habits is considered obesogenic (20).

According to the objective of this research, a sub-pattern of food consumption was found that would allow differentiating between the preferences of those with euglycemia and those with prediabetes and diabetes values. The factorial analysis revealed a marked difference in the dietary patterns, mainly in the larger limitation of chosen food, which were those with the highest concentration of saturated fats and industrialized sugars (Table 3).

**Table 3:** Analysis of component matrices

	Fasting glucose level		
	
Component	Euglycemia<5.5 mmol/L	Prediabetes 5.6–6.9 mmol/L	Diabetes ≥7 mmol/L
First	Red meats 0.799	Cold cuts 0.750	Cold cuts 0.829
	Cold cuts 0.750	Pastas 0.732	Fatty food 0.749
	Fried food 0.715	Red meats 0.696	Fried food 0.699
	White bread 0.662	White bread 0.682	White bread 0.618
	Pastas 0.655	Sugar-added 0.669	
	Pastry 0.636	Pastry 0.652	
	Fatty food 0.632	Breakfast cereals 0.634	
	Sugar-added 0.609		
Second	Hot food 0.668	Hot food 0.671	Fruits 0.797
	Leguminose 0.655	Dressings 0.670	Cereals 0.755
		Vegetables 0.619	Juices 0.678
		Tubercles 0.606	Dairy 0.613
Third	Eaten fruits 0.735	Juices 0.649	Breakfast cereals 0.813
	Juices 0.603	Legumes 0.614	Added sugar 0.643
	Cereals 0.6		Alcoholic beverages 0.628
			Pastry 0.604

Extraction method: principal components analysis. Rotation method: Varimax with Kaiser normalization.

Many studies on food address overweight–obesity (21–23), focusing mostly on the dietary patterns rather as an aesthetic than a health concern. However, the change in eating habits, toward the predominance of modern industrialized, has been associated with the possibility of presenting metabolic anomalies, like insulin resistance (24), type 2 diabetes mellitus (25), or liver steatosis (26), independently of whether obesity is generated before or after presenting metabolic anomalies. The anomalies found in the biochemical markers independently from the body mass, but associated with dietary patterns, strengthen the study. Another important finding is the high proportion of participants with anomalies since they are in an intermediate stage between adolescence and adulthood.

### Limitations

As in all research dependent on the responses of the participants, involuntary bias can occur when recalling the eating habits even of just 1 month. A limiting aspect is that, despite the correlation, no cause-and-effect associations can be made. Another limitation is the cross-sectional design of the study. Although the studied population corresponded to young adults of a determined geographical zone, the possibility of genetic variations cannot be dismissed.

## Conclusions

A sub-pattern of food consumption correlated with fasting glucemia ≥5.6 mmol/L was found. Despite that the study population corresponds to young adults starting their university education, no association seems to exist between schooling level and the capacity to choose a healthy diet. This is based on the finding that the population with euglycemia also consumes a diet considered obesogenic; hence, it is quite likely that, in the mid-term, a higher proportion of young adults will be found with anomalies in their biochemical markers. Data support the need to elaborate educational processes to encourage pre-university students to choose their food according to a healthier lifestyle.
